# *Bifidobacterium*-Escherichia coli Shuttle Vector Series pKO403, with Temperature-Sensitive Replication Origin for Gene Knockout in *Bifidobacterium*

**DOI:** 10.1128/mra.00884-21

**Published:** 2022-01-13

**Authors:** Hend Altaib, Yuka Ozaki, Tomoya Kozakai, Kouta Sakaguchi, Izumi Nomura, Tohru Suzuki

**Affiliations:** a The United Graduate School of Agricultural Science, Gifu University, Gifu, Japan; b Faculty of Applied Biological Sciences, Gifu University, Gifu, Japan; Indiana University, Bloomington

## Abstract

A series of *Bifidobacterium*-Escherichia coli shuttle vectors (pKO403-*lacZ′*-Cm, pKO403-*lacZ′*-Sp, pKO403-*lacZ′*-p15A) were constructed based on the pKO403 backbone, which carries a temperature-sensitive replication origin. These vectors carry the *lacZ′*α fragment, overhung by two facing type IIS restriction sites, for blue-white selection and seamless gene cloning. These vectors are useful for gene knockout or multigene integration into the chromosome of *Bifidobacterium*.

## ANNOUNCEMENT

Plasmid vectors for gene expression, manipulation, and gene knockout are fundamental tools for the metabolic engineering of bacteria (1). Techniques such as seamless gene cloning and white-blue selection have been developed to facilitate the cloning process (2, 3). Vector pKKT427 was constructed for gene expression in bifidobacteria (4). It is highly efficient at transformation in *Bifidobacterium*. pKO403 is a shuttle vector derived from pKKT427. It is a temperature-sensitive vector, which is cured at 42°C. It is a very powerful tool to produce gene knockout mutants of *Bifidobacterium* (5). However, it still has limited cloning features. For instance, it is not specialized for seamless gene cloning, which is required for precise assembly of multiple DNA fragments, as it leaves no additional sequences between the ligated fragments. In order to expand the cloning features of pKO403, this announcement describes three Bifidobacterium-Escherichia coli shuttle vectors based on the pKO403 backbone to facilitate genetic manipulations of *Bifidobacterium*, such as gene expression and gene knockout. Here, we present new vectors that enable the seamless ligation of multiple DNA fragments into the pKO403 backbone using the Golden Gate cloning technique. The alternative pKO403 series was constructed as follows.

The *lacZ′* gene, which encodes the α fragment of the E. coli *lacZ* gene, was amplified from pUC18 (forward, gcctaggcctgtcgaaca**tgaagagc**GTCACAGCTTGTCTGTAAGC; reverse, gccctgagggcggcccta**cgaagagc**GCCCAATACGCAAACC; the underlined lowercase characters are the In-Fusion tag, and the bold sequences indicate the SapI restriction site). Two SapI restriction sites were added to both the forward and reverse sequences of *lacZ′.* The amplified sequence was inserted into pKO403 using the In-Fusion HD cloning kit (TaKaRa, Tokyo, Japan). SapI restriction sites in the backbone of pKO403 were removed by site-directed mutagenesis, producing pKO403*-lacZ′*-Sp using the following primers (the modified nucleotide is in lowercase): forward, gTCTCACATCAGAAAATGG; reverse, GAGCCATTATGGATTCGT. In order to allow a broad range of antibiotic sensitivity vectors, pKO403-*lacZ′*-Cm was constructed by replacing the spectinomycin resistance gene (Sp^r^) with the chloramphenicol resistance gene (*cat*). The *cat* gene was amplified from pUC18 carrying the *cat* gene and *hup* promoter. The amplification primers were as follows (the underlined lowercase characters are the In-Fusion tag): forward, gtatatatgagtactTGGGCGCGGCGGCCATGAA; reverse, ctcagtactctgcagTGGAACGTAATAAAAAAAGCGGGCTC. A linear plasmid backbone without Sp^r^ was amplified from pKO403*-lacZ′*-Sp using the following primers: forward, CTGCAGAGTACTGAGCT; reverse, AGTACTCATATATACTTTAGATT. Both fragments were ligated using In-Fusion.

To improve plasmid stability in *Bifidobacterium*, the ColE1 replication origin in pKO403*-lacZ′*-Sp was replaced with p15A. The primers were designed to amplify both the p15A region from pBAD33 (forward, ccagctcttcgATGAGCGGAGTGTATACTGGC; reverse, ccagctcttcgTACTTCGGGCTCATGAGCAA; the underlined characters indicate the SapI restriction site) and the plasmid backbone, without the replication region ColE1, from pKO403*-lacZ′*-Sp (forward, CCAgctcttcGTAGTATATATGAGTACTGAATTCCC; reverse, CCAgctcttcGCATCTCCGCTGAATTCGAG; the underlined characters indicate the SapI restriction site). The two PCR products were ligated using Golden Gate cloning (6), producing pKO403*-lacZ′-*p15A.

**FIG 1:**
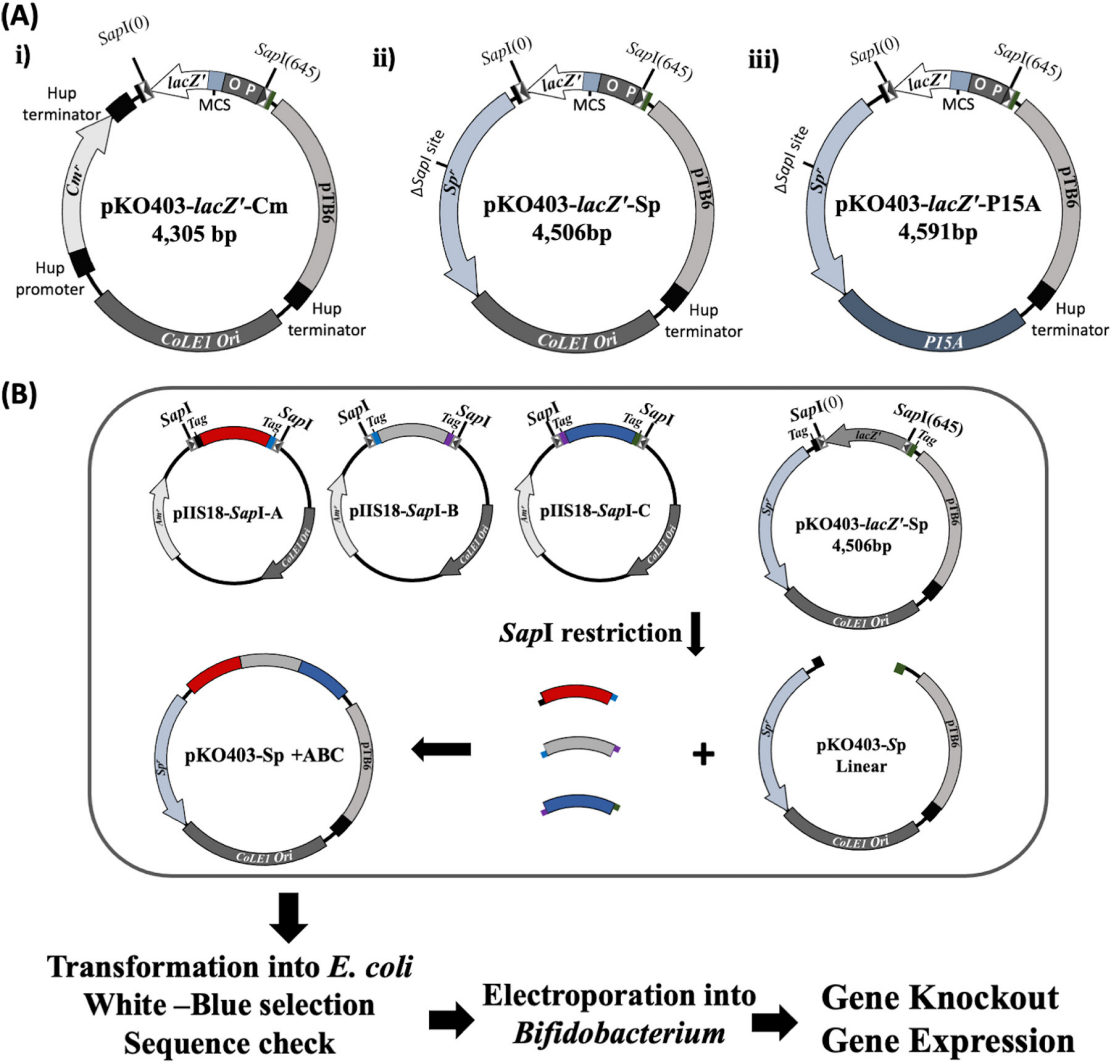
(A) Molecular structure of vector series pKO403, demonstrating structures on the backbone. (i) pKO403-*lacZ′*-Cm, carrying the *lacZ′* gene with promoter (P), operator (O), multiple cloning site (MCS), chloramphenicol resistance gene (Cm), ColE1 origin of replication, and pTB6 region for temperature sensitivity. (ii) pKO403-*lacZ′*-Sp, carrying a similar structure to pKO403-*lacZ′*-Cm, except for replacing the chloramphenicol resistance gene with the spectinomycin resistance gene (Sp). (iii) pKO403-*lacZ′*-p15A, carrying the p15A origin of replication. (B) Model for the usage of vectors pKO403 and pIIS18 for Golden Gate cloning. It demonstrates the possibility of using pKO403 with other entry vectors carrying type IIS recognition sites, with matching tags, for multifragment joining.

The constructed plasmids were introduced into E. coli TOP10, and the resulting transformants were selected on an LB agar plate with the respective antibiotic (spectinomycin [Sp], chloramphenicol [Cm]) and 2% X-Gal (5-bromo-4-chloro-3-indolyl-β-d-galactopyranoside). The blue colonies were subcultured in LB broth, and the plasmid was extracted using a Qiagen miniprep kit. A whole-plasmid sequence was performed using the BigDye Terminator version 3.1 cycle sequencing kit. The sequence data were analyzed using an ABI3130xl genetic analyzer (Thermo Fisher Scientific, Inc.). The three vectors are displayed in Fig. 1A.

The introduced vector series are useful for molecular cloning in both E. coli and *Bifidobacterium*. Using pKO403-*lacZ*′-Sp and pKO403-*lacZ*′-Cm, multifragment ligation for gene expression and gene knockout can be used with precision. We previously constructed an E. coli vector series, pIIS18, for seamless gene cloning (7). Using the pKO403*-lacZ*′ vectors with pIIS18 vectors enables the application of the Golden Gate strategy in multigene cloning and gene knockout. The matching tags in both the insert and pKO403 give the advantage of seamless gene fusion (Fig. 1B). The temperature sensitivity of the pKO403 series allows the plasmid to replicate at 37°C, while it will be unstable at higher temperatures (5). Therefore, only the recombinants, carrying a marker gene such as *cat* or Sp^r^, can grow on MRS plates containing the corresponding antibiotic at 42°C. Using this plasmid, it is easy to select the double-crossover recombinants leading to gene knockout or gene knock-in of bifidobacteria (5). p15A was reported to improve plasmid stability in bacteria (8–10). Therefore, pKO403-*lacZ*′-p15A paves the way for stable long-acting gene expression.

### Data availability.

The complete sequences of plasmids pKO403-*lacZ*'-Cm, pKO403-*lacZ*'-Sp, and pKO403-*lacZ*'-p15A have been deposited at the DNA Data Bank of Japan under the accession numbers LC647191, LC647192, and LC647193, respectively. The resources are available from the Addgene depository (https://www.addgene.org/). The raw sequencing reads are available at https://www1.gifu-u.ac.jp/~suzuki/pKO_plasmids/.
